# Dynamics and hydrodynamic efficiency of diving beetle while swimming

**DOI:** 10.1016/j.heliyon.2023.e14200

**Published:** 2023-03-01

**Authors:** Debo Qi, Chengchun Zhang, Zhengyang Wu, Chun Shen, Yongli Yue, Luquan Ren, Liang Yang

**Affiliations:** a Key Laboratory of Bionic Engineering (Ministry of Education), Jilin University, Changchun, 130025, China; b Weihai Institute for Bionics, Jilin University, Weihai, 264402, China; c State Key Laboratory of Automotive Simulation and Control, Jilin University, Changchun, 130025, China; d Lecturer in Marine Renewable Energy System, Cranfield University, Cranfield, MK43 0AL, UK

**Keywords:** Diving beetle, Propulsion force, Drag force, Added mass force, Hydrodynamic efficiency

## Abstract

Diving beetle, an excellent biological prototype for bionic underwater vehicles, can achieve forward swimming, backward swimming, and flexible cornering by swinging its two powerful hind legs. An in-depth study of the propulsion performance of them will contribute to the micro underwater vehicles. In this paper, the kinematic and dynamic parameters, and the hydrodynamic efficiency of the diving beetle are studied by analysis of swimming videos using Motion Capture Technology, combined with CFD simulations. The results show that the hind legs of diving beetle can achieve high propulsion force and low return resistance during one propulsion cycle at both forward and backward swimming modes. The propulsion efficiencies of forward and backward swimming are 0.47 and 0.30, respectively. Although the efficiency of backward swimming is lower, the diving beetle can reach a higher speed in a short time at this mode, which can help it avoid natural enemies. At backward swimming mode, there is a long period of passive swing of hind legs, larger drag exists at higher speed during the recovery stroke, which reduces the propulsion efficiency to a certain extent. Reasonable planning of the swing speed of the hind legs during the power stroke and the recovery stroke can obtain the highest propulsion efficiency of this propulsion method. This work will be useful for the development of a bionic propulsion system of micro underwater vehicle.

## 1 Introduction

Diving beetles, which belong to the Insecta, Coleoptera, and family Dytiscidae, are aquatic creatures that swim in the water by synchronously paddling the right and left hind legs [1,2]. Their swimming ability is astonishing, for which they can skillfully carry out quick and slow forward swimming, quick retreating swimming, and flexible turning swimming [[3], [4], [5], [6], [7]], and these characteristics have caused the interest of many researchers. Diving beetles have three pairs of legs: a pair of forelegs used primarily for feeding and grabbing objects, a pair of middle legs used to stabilize the body during the recovery stroke while swimming, and a pair of hind legs used to generate propulsion force for movement [8]. Studies show that the hind legs can generate a larger angular velocity than the middle legs [9]. The hind leg consists of three segments, which are the femur, tibia, and tarsus [[10], [11], [12]]. A large number of swimming hairs are distributed on the inner and outer edges of the tarsus, and they are the main source of forward thrust force [11,[13], [14], [15], [16]]. Body shapes of them are streamlined, which can help reduce the drag of water and improve swimming stability [12]. Their propulsion method, synchronously paddling the hind legs, has been proven that have higher hydrodynamic efficiency than alternating-leg-swimming kinematics [2]. Inspired by the stroke propulsion of the diving beetle, some researchers have carried out research on bionic robots, such as two bio-inspired robots with a pair of legs with swimming appendages (one with hair-like appendages and the other with flaky appendages) [10,17]. However, the swimming appendages were simplifications of the swimming hairs, which could not fully reflect the actual swimming characteristics of diving beetles. Especially during the recovery stroke, the backward phenomenon occurred with all robots, which reflected the considerable amount of resistance with the swimming appendages. A bio-inspired multi-functional legged robot that mimicked the diving beetle was also designed [18]. Although it could complete swimming motion underwater, its turning efficiency was much lower compared with the real diving beetle. The robot required five strokes to complete a left or right turning motion, while the diving beetle only needed one stroke. Another study examined a robot propelled by an alternating magnetic field that mimicked the diving beetle [3,19]. The experiments showed that the difference in the swimming numbers (how many body lengths per beat to swim) between the robot and the diving beetle was huge, 0.07 and 0.98, respectively.

For the design of underwater robots that mimic diving beetles, it is necessary to conduct in-depth research on the dynamic characteristics of the diving beetle while swimming underwater. This paper focuses on the propulsion force, return resistance, and propulsion efficiency of diving beetles while swimming forward or backward along an approximate straight line. This work reveals the mechanical properties of diving beetles and provides a bionic foundation for the design of the micro underwater vehicle.

## 2 Results

### 2.1 The kinematic parameters

**Fig. 1:**
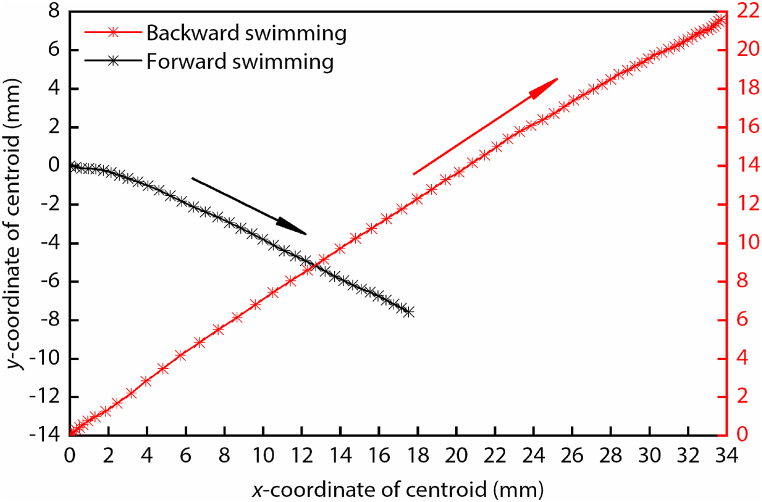
The coordinates of the centroid of the diving beetle during straight forward swimming and straight backward swimming. The maximum coordinate errors for forward swimming and backward swimming are 192 μm and 272 μm, respectively.

As shown in Fig. 1, the trajectories of the centroid of the diving beetle during straight forward and backward swimming maintain a straight line. From this, we know that the diving beetle can maintain linearity during the forward and backward swimming. The displacement in the *y* direction during backward swimming is about three times that of forward swimming, and the displacement in the *x* direction during backward swimming is about twice that of forward swimming. The total displacement during backward swimming is about twice that of forward swimming.

**Fig. 2:**
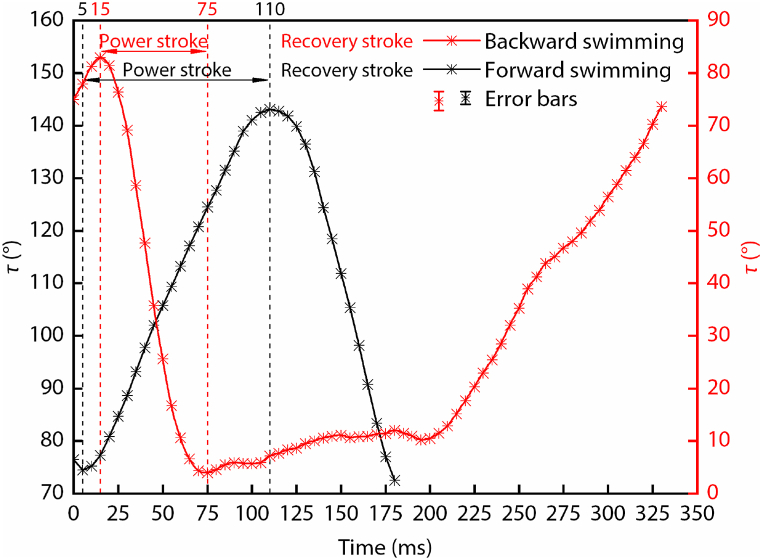
Curves of the angle *τ* change with time during straight forward swimming and straight backward swimming.

Based on the swimming videos of the diving beetle, we tracked the angle *τ* between the femur of the hind leg and the diving beetle’s body axis (as shown in Fig. 2). To make it easy to distinguish between the power stroke and recovery stroke, we selected the time point where *τ* changes from increasing to decreasing or from decreasing to increasing as the distinguishing sign. For straight forward swimming (the black curve), the diving beetle achieves its power moving forward by swinging its hind legs backward (power stroke), and then by swinging the hind legs forward to prepare for the next cycle (recovery stroke). So the angle *τ* keeps increasing during the power stroke and decreasing during the recovery stroke. For straight backward swimming (the red curve), everything is opposite. The period of the power stroke during forward swimming is 105 ms, which is nearly twice as long as that of backward swimming (60 ms), and the swing angles of the femur are about 70° and 80°, respectively. All of this proves that straight backward swimming can help the diving beetle obtain higher acceleration and speed, which is beneficial for avoiding natural enemies. Conversely, the diving beetle needs more time to finish the recovery stroke (270 ms) during backward swimming, which is nearly four times as long as that of forward swimming (75 ms). During backward swimming, after the power stroke is over, the angle *τ* starts to increase, but it is not obvious, and the angle *τ* changes slowly over a long period of time. At the end of the recovery stroke, the angle *τ* starts to increase rapidly. The longer period of the recovery stroke will reduce the efficiency of propulsion.

**Fig. 3:**
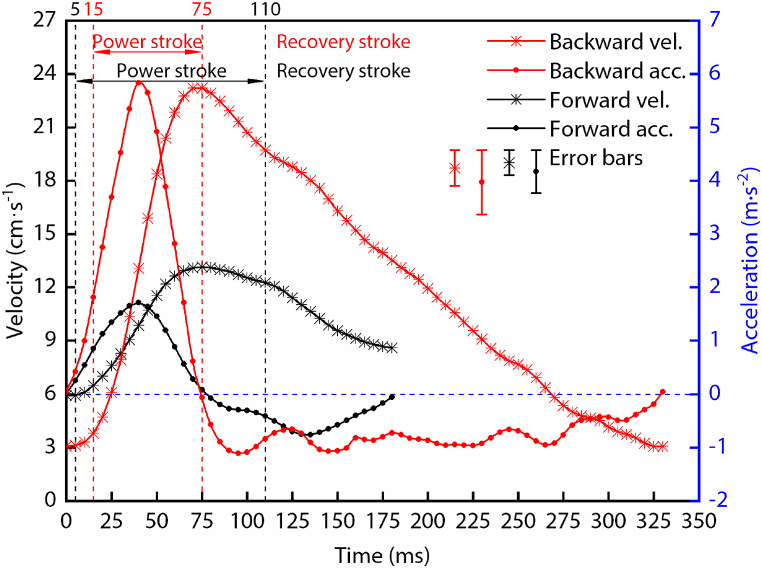
The speed and acceleration of the diving beetle during straight forward swimming and straight backward swimming. Where “vel.” and “acc.” are the abbreviations of velocity and acceleration, respectively.

As depicted in Fig. 3, the velocities of the diving beetle during forward and backward swimming all reached their peak at 75 ms, and the accelerations all reached their peak at 40 ms. Based on the time period of the power stroke of the two swimming motions, 40 ms was about at the halfway point of the power stroke during backward swimming, which was also about at one-third of the power stroke during forward swimming. The acceleration process ended when the hind legs completed the forward swing during backward swimming, i.e., the diving beetle kept accelerating during the total process of the forward swing of the hind legs. Whereas at two-thirds of the backward swing of the hind legs during forward swimming, the diving beetle began decelerating, i.e., the propulsion force of the hind legs was already less than the resistance. All of these reflect that the diving beetle keeps a higher acceleration efficiency during backward swimming. The peak values of the velocity and acceleration during backward swimming are about two times and four times that of during forward swimming, respectively. This shows that the diving beetle has a more explosive power during backward swimming.

### 2.2 The kinetic parameters

**Fig. 4:**
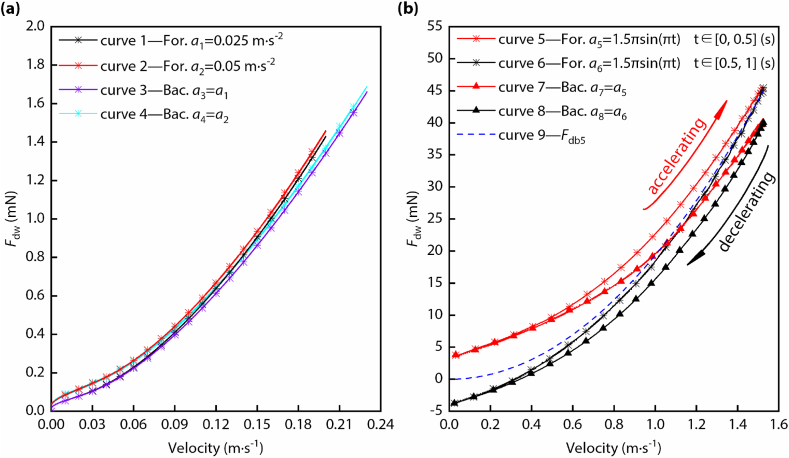
The simulation results of the acceleration processes of the diving beetle. (a) The curves of the drag of water when the accelerations are constant. (b) The curves of the drag of water when the accelerations change sinusoidally. The meanings of “For.” and “Bac.” are forward swimming and backward swimming, respectively.

The simulation results of the acceleration processes of the diving beetle are as shown in Fig. 4. By comparing curves 1 and 2 (or curves 3 and 4), we found that they were parallel, from which we could conclude that the added mass force and added mass are stable under the condition that the acceleration is constant during the swimming motion. Curves 1 and 3 (or curves 2 and 4) have the same acceleration, but the movement directions of the diving beetle are opposite (*a*_1_ = *a*_3_; *a*_2_ = *a*_4_; *a*_1_ ≠ *a*_2_). When the velocity got smaller, the curves gradually overlapped and gradually separated with the increase of velocity. Based on the calculation formula of *F*_dw_ (Eq. (16)), when the velocity was small, the quadratic term of velocity could be left out, leaving only the linear term of velocity and the added mass force term. Although the accelerations of the two curve groups (group 1: curves 1 and 3; group 2: curves 2 and 4) were different, all curves in each group had overlapped finally. This reflects that coefficients $\hat{b}$ and $\hat{c}$ for forward swimming are very close to the corresponding coefficients for backward swimming. When the velocity got bigger, the curves in each group gradually separated. This is because the coefficient $\hat{a}$ in the calculation formula of *F*_dw_ is different for forward swimming and backward swimming (as shown in Fig. 4(a) and Table 1). Curves 5–8 are the drag of water when the acceleration changed sinusoidally (as shown in Fig. 4(b)). Curves 5 and 7 are the drag of water when the diving beetle accelerated forward and backward, respectively, and curves 6 and 8 are the drag of water when the diving beetle decelerated forward and backward. From curve 5 and curve 7, we could find that the curves overlapped at the beginning of the accelerations and separated at the end of the accelerations, which reflects that the added mass force or added mass of the two acceleration processes are close and the drag force of the diving beetle’s body while accelerating forward is bigger than that when accelerating backward. Curve 9 is the drag force of the diving beetle’s body while accelerating forward, and if there is no added mass force during the deceleration process of the diving beetle, the curve of the water drag force during the deceleration process will be curve 9. The result was that curve 6 was the water drag force, which proved that the added mass force does exist during the deceleration process. Since curve 6 was below curve 9, the direction of the added mass force was the diving beetle’s movement direction. Curve 6 and curve 8 overlapped at the end of the decelerations, which reflects that the added mass force or added mass of the two deceleration processes are close (as shown in Table 2).

**Table 1:** The fitting results of curves 1–8 in Fig. 4

Number *i*	Motion modes	Acceleration *a*_*i*_ (m·s^−2^)	velocity range (m·s^−1^)	$F_{\text{dw}}=\hat{a}v^{2}+\hat{b}v+\hat{c}a$ (mN)				$F_{\text{dw}}=\hat{d}v^{2}+\hat{e}a$ (mN)			added mass m* (g)
				${\hat{a}}_{i}$	${\hat{b}}_{i}$	${\hat{c}}_{i}$	R^2^	${\hat{d}}_{i}$	${\hat{e}}_{i}$	R^2^	
1	forward	0.025	[0, 0.2]	25.00	2.086	0.9256	0.9998	34.85	3.994	0.9946	$m^{*}={\hat{c}}_{1}=0.9256$
2	forward	0.05	[0, 0.2]	24.93	2.104	1.074	0.9998	34.69	2.681	0.9945	$m^{*}={\hat{c}}_{2}=1.074$
3	backward	0.025	[0, 0.23]	20.62	2.422	0.8814	0.9995	30.89	4.78	0.9924	$m^{*}={\hat{c}}_{3}=0.8814$
4	backward	0.05	[0, 0.23]	20.63	2.488	0.9143	0.9996	30.76	3.062	0.9925	$m^{*}={\hat{c}}_{4}=0.9143$
5	forward	$a_{5}$	[0.306, 1.525]	21.49	−3.482	1.322	0.9996	19.24	1.026	0.9995	$m^{*}={\hat{e}}_{5}=1.026$
6	forward	$a_{6}$	[1.525, 0.306]	17.73	2.679	0.5321	0.9999	19.45	0.3042	0.9999	$m^{*}={\hat{e}}_{6}=0.3042$
7	backward	$a_{7}$	[0.306, 1.525]	22.38	−8.572	1.69	0.9986	16.85	0.9608	0.9976	$m^{*}={\hat{e}}_{7}=0.9608$
8	backward	$a_{8}$	[1.525, 0.306]	17.08	0.052	0.4198	0.9999	17.12	0.4145	0.9999	$m^{*}={\hat{e}}_{8}=0.4145$

The velocities for number 5 and number 7 are as Eq. (21), and the velocities for number 6 and number 8 are as Eq. (22).

*v* = 1.5 × sin(π*t*) + 0.025 0.06 ≤ *t* ≤ 0.5 (21)

*v* = 1.5 × sin(π*t*) + 0.025 0.5 ≤ *t* ≤ 0.94 (22)

**Table 2:** The values of kinetic parameters of the diving beetle.

Motion modes	*v* (m·s^−1^)	*S*_*W*_ (10^−4^ m^2^)	*C*_1_	*C*_2_	m* (g)	*F*_dw_ = *F*_db_ + *F*_am_ (mN)
Acc. forward	[0, 0.2]	1.667	72.20	0.30	1.026	2.09*v*+24.97*v*^2^+1.026*a*
Dec. forward	[0, 0.2]	1.667	72.20	0.30	0.3042	2.09*v*+24.97*v*^2^+0.3042*a*
Acc. backward	[0, 0.23]	1.667	84.98	0.25	0.9608	2.46*v*+20.63*v*^2^+0.9608*a*
Dec. backward	[0, 0.23]	1.667	84.98	0.25	0.4145	2.46*v*+20.63*v*^2^+0.4145*a*

The values of $\hat{a}$, $\hat{b}$, and $\hat{c}$ were obtained by fitting curve 1 to curve 8, and the results were listed in Table 1. Based on Eqs. (17), (18), (20), the values of m*, *C*_1_, and *C*_2_ were listed in Table 2, and based on the fitting results, *F*_dw_ was obtained.

For the purpose of illustrating the accuracy of the simulation process, the acceleration process of an ellipsoid was simulated (as shown in Supplementary Data S1). Three axes of the ellipsoid were 36 mm, 16 mm, and 16 mm (determined by the formula sqrt(21*12)), similar to the size of the diving beetle (36 mm, 21 mm, 12 mm). According to Ref. [20], under the condition that the translation direction is along the long axis, the added mass of the ellipsoid could be calculated by Eq. (1). The constant *α*_0_ in Eq. (1) could be calculated by Eq. (2).

(1) $$m^{*}=\frac{\alpha_{0}}{2-\alpha_{0}}\times \frac{4}{3}\rho \pi ab^{2}$$

(2) $$\alpha_{0}=\frac{1-e^{2}}{e^{3}}\left(\ln\frac{1+e}{1-e}-2e\right)$$

where m* is the added mass of ellipsoid; *ρ* is the density of water; *a* and *b* are the major and minor axes of the ellipsoid, respectively; *e* is the eccentricity of sections through the axis of symmetry.

The simulation results showed that the added mass during the acceleration process was 0.8928 g, and the added mass during the deceleration process was 0.8514 g. The theoretical value of the added mass of the ellipsoid was 0.8679 g and the relative errors were 2.9% and 1.9%, respectively. Therefore, the simulation process is reliable.

**Fig. 5:**
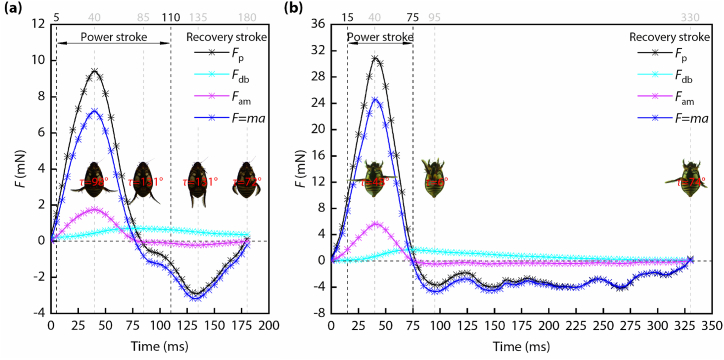
Force analysis results of the diving beetle while swimming. (a) The propulsion force of the hind legs (*F*_p_) and the forces of its components during forward swimming. (b) The propulsion force of the hind legs (*F*_p_) and the forces of its components during backward swimming.

Based on Eqs. (6), (7), it is easy to calculate *F*_p_ and *F*_dl_ after these kinematic and kinetic parameters have been calculated (as shown in Fig. 5). During forward swimming (as shown in Fig. 5(a)), the time period when the propulsion force was greater than zero accounted for about 77% of the power stroke. At 40 ms, the propulsion force reached its peak value, and the hind legs were fully extended and swung backward at this time. From 85 ms to 110 ms, the propulsion force was less than zero, i.e., during this period of time, the forces output by the hind legs were drags. 25 ms after the power stroke was over, the drag force of the hind legs (*F*_dl_) reached its peak value, and the swimming hairs pointed toward the body axis under the pressure of water. The ratio between the peak values of *F*_p_ and *F*_dl_ was about 3. At the end of the entire cycle, the drag force of the hind legs (*F*_dl_) and the inertial force (*F* = *ma*) were roughly equal.

During backward swimming (as shown in Fig. 5(b)), the propulsion force was greater than zero throughout the power stroke due to the rapid forward swing of the hind legs. Similar to forward swimming, 20 ms after the power stroke was over, the drag force of the hind legs also reached its peak value, and the swimming hairs pointed toward the body axis under the pressure of water. This shows that during the transition from power stroke to recovery stroke, the swimming hairs help the drag of the hind legs reach the peak. The ratio between the peak values of *F*_p_ and *F*_dl_ was about 8. During the entire recovery stroke, the drag force of the hind legs was stable. At the end of the recovery stroke, the drag force of the hind legs (*F*_dl_) and the inertial force (*F* = *ma*) were roughly equal, i.e., the drag force of the body (*F*_db_) and the added mass force (*F*_am_) were negligible.

**Fig. 6:**
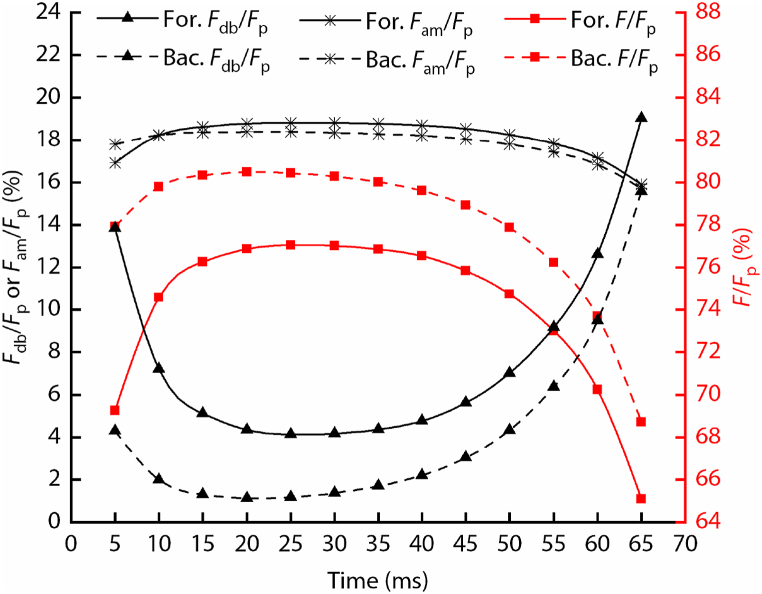
The percentage of each component of the propulsion force of the hind legs during the power stroke. The meanings of “For.” and “Bac.” are forward swimming and backward swimming, respectively.

As shown in Fig. 6, during most time of the power stroke of forward swimming, the forces *F*_db_, *F*_am_, and *F* accounted for about 4%, 19%, and 77%, respectively, and their values changed to 1.5%, 18.5%, and 80% during backward swimming. Within the time range on both sides of the power stroke, since the acceleration of the diving beetle was small, the percentage of *F*_db_ had a maximum increase of 15%, and the percentage of *F*_am_ and *F* had a maximum decrease of 3% and 12%, i.e., the percentage of *F*_am_ was stable during the power stroke. These values reflect that the drag force of the body (*F*_db_) only occupies a small part, the inertial force (*F* = *ma*) occupies the majority, and the added mass force (*F*_am_) is not negligible.

**Fig. 7:**
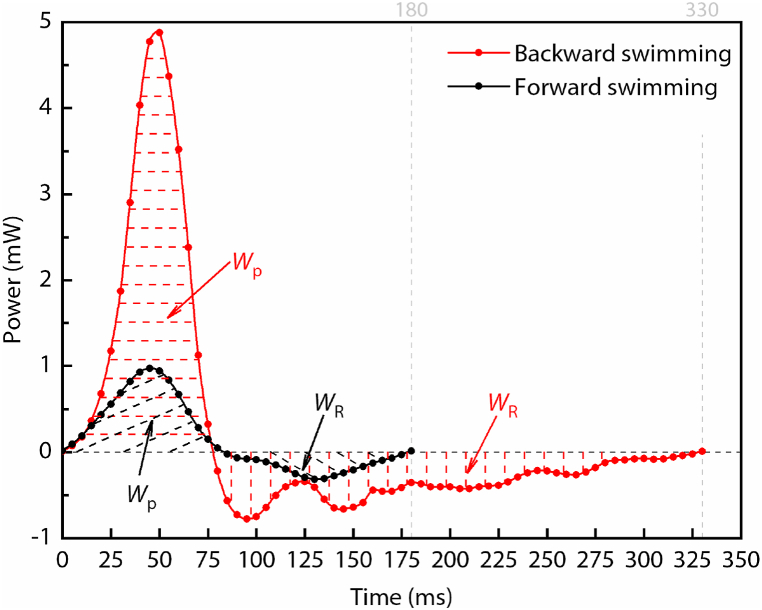
The power of the force produced by the hind legs during forward swimming and backward swimming. *W*_p_ is the work done by the propulsion force during the power stroke, and *W*_R_ is the work done by the drag force during the recovery stroke.

In order to obtain the hydrodynamic efficiency of the diving beetle, we calculated the power of the force produced by the hind legs during forward swimming and backward swimming by using Eq. (3) (as shown in Fig. 7). The work done by the propulsion force during the power stroke (*W*_p_, as shown in Fig. 7) and the work done by the drag force of the hind legs during the recovery stroke (*W*_R_, as shown in Fig. 7) are calculated by Eq. (4). As a propulsion factor, the direction of the propulsion force is the same as that of the diving beetle’s movement, while the drag force is opposite. Therefore, only *W*_p_ is used for the diving beetle’s body propulsion, while *W*_R_ will reduce the kinetic energy of the diving beetle. So the work that is useful for the motion of the diving beetle should be *W*_p_-*W*_R_, and the ratio of this part to the total work (*W*_p_ + *W*_R_) done by the hind legs during the entire cycle is the efficiency of underwater swinging as a propulsive factor, which could be calculated by Eq. (5).

(3) $$P=F_{p}\left(t\right)v\left(t\right)\;\text{or}\;P=F_{\text{dl}}\left(t\right)v\left(t\right)$$

where *F*_p_(*t*) is the propulsion force of the hind legs at the moment of *t* during the power stroke; *F*_dl_(*t*) is the drag force of the hind legs at the moment of *t* during the recovery stroke; *v*(*t*) is the velocity of the diving beetle at the moment of *t*.

(4) $$\left\{ \begin{array}{l} W_{p}=\int_{0}^{T_{p}}\left(F_{p}\left(t\right)v\left(t\right)\right)dt\approx \sum_{i=1}^{n}\frac{\left(F_{p}\left(t_{i}\right)v\left(t_{i}\right)+F_{p}\left(t_{i+1}\right)v\left(t_{i+1}\right)\right)T_{p}}{2n}\\ W_{R}=\int_{T_{p}}^{T_{p}+T_{R}}\left(F_{\text{dl}}\left(t\right)v\left(t\right)\right)dt\approx \sum_{i=1}^{m}\frac{\left(F_{\text{dl}}\left(t_{i}\right)v\left(t_{i}\right)+F_{\text{dl}}\left(t_{i+1}\right)v\left(t_{i+1}\right)\right)T_{R}}{2m} \end{array}\right.$$

where T_p_ is the period of the power stroke; T_R_ is the period of the recovery stroke; *n* and *m* are the numbers that equally divide the power stroke and the recovery stroke, respectively, and we control $T_{p}/n=T_{R}/m=5\;\text{ms}$.

(5) $$\eta =\frac{W_{P}-W_{R}}{W_{P}+W_{R}}$$

Based on the calculation results, the values of *W*_p_, *W*_R_, and *η* during forward swimming are 0.042 mJ, 0.015 mJ, and 0.47, and their values change to 0.163 mJ, 0.088 mJ, and 0.30 during backward swimming. Although the value of *W*_p_ is almost four times that of forward swimming, due to the long period of the recovery stroke, the value of *W*_R_ is almost six times that of forward swimming, which results in an efficiency that is 0.17 lower than that of forward swimming. If we look at Fig. 2, we will find that from 75 ms to 200 ms, the value of *τ* only increases by 5°. This means that the diving beetle is basically in a state of passively swinging its hind legs, which seriously affects its propulsion efficiency. If this time period is removed, the period of the recovery stroke is reduced by 125 ms, and the total cycle of backward swimming becomes 205 ms, which is only 25 ms longer than that of forward swimming, and the propulsion efficiency changes to 0.71. From the analysis of the propulsion efficiency of the forward and backward swimming, even though the swing velocity of the diving beetle’s hind legs during forward swimming is less than that of backward swimming, the propulsion efficiency is higher, and the efficiency of backward swimming is not as high as expected.

## 3 Discussion

The kinematic parameters analysis results showed that the velocities and accelerations of diving beetle during forward and backward swimming all reach their peaks at 75 ms and 40 ms, respectively, which demonstrates that the diving beetle keeps accelerating during the entire power stroke of backward swimming. Conversely, during the last 35 ms of the power stroke of forward swimming, the diving beetle keeps decelerating. The peak values of velocity and acceleration during backward swimming are about two times and four times that of forward swimming, respectively. This shows that the diving beetle has more explosive power during backward swimming.

During forward swimming, the time period that the propulsion force is greater than zero accounts for about 77% of the power stroke. Whereas, the propulsion force is greater than zero throughout the power stroke of backward swimming, which benefits from the rapid forward swing of the hind legs. The ratio of the peak value of *F*_p_ to *F*_dl_ is about 3 and 8, respectively, and the ratio of the peak value of *F*_p_ during forward swimming to the peak value of *F*_p_ during backward swimming is about 3. During most time of the power stroke of forward swimming, the forces *F*_db_, *F*_am_, and *F* account for about 4%, 19%, and 77%, respectively. These values change to 1.5%, 18.5%, and 80% during backward swimming. Thus the percentage of the force used to accelerate the diving beetle is approximately 77%–80%.

The propulsion efficiency of backward swimming is 0.30, which is 0.17 lower than that of forward swimming. The reason is that there is a long period of passive swing of hind legs during the propulsion cycle. If this time period is removed, the propulsion efficiency changes to 0.71. Although the swing speed of the diving beetle’s hind legs during forward swimming is less than that of backward swimming, the propulsion efficiency is higher. The advantage of backward swimming is that it can help the diving beetle reach a higher speed in a short time, which can help it avoid natural enemies. But the higher speed also leads to a higher drag during the recovery stroke, which will lead to a reduction in the propulsion efficiency. Therefore, reasonable planning of the swing speed of the hind legs during the power stroke and the recovery stroke can obtain the highest propulsion efficiency of this propulsion method.

## 4 Materials and methods

### 4.1 Diving beetles

In this paper, we selected *Cybister bengalensis* as the research object. The diving beetles used in the experiments were adults captured at Foshan City (Guangdong Province, China), all about the same size. The physical parameters of the body and the three segments of the hind legs (femur, tibia, and tarsus) are shown in Table 3. The longest one among the three segments is the tarsus, which consists of 5 sub-sections. The tibia is the shortest segment, about half the length of the femur. These characteristics of the hind legs were considered to be an adaptation of high-speed swimming [21]. Numerous swimming hairs with diameters ranging from 5 to 20 μm are distributed on the inner and outer edges of the 5 sub-sections of the tarsus. All features mentioned above are helpful for swimming [22,23].

**Table 3:** Physical parameters of the diving beetles.

Number	Body (mm)	mass (g)	Femur (mm)	Tibia (mm)	Tarsus (mm)					
					Total	1	2	3	4	5
1	37	4.3	9.5	4.6	14.5	4	2.5	2	2	4
2	36	4.2	9	4.7	13.1	3.3	2.1	2	1.7	4
3	35.5	4.0	10	5.5	13.2	3.5	2	1.9	1.8	4
4	36	4.1	9.6	4.5	12.8	3	2	2	1.8	4
5	37	4.2	9.8	4.8	12.7	3	2	1.8	1.9	4

### 4.2 Experimental apparatus

**Fig. 8:**
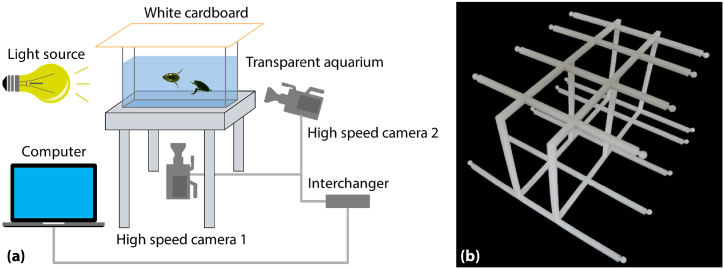
Instruments and methods. (a) Schematic diagram of experimental methods: a transparent glass water tank was placed on the transparent desktop, an LED lamp served as a light source, and the swimming videos were synchronously captured by two high-speed cameras from the bottom and side of the glass water tank, which were recorded by the computer that connected with the two cameras. (b) The calibration frame for coordinate system establishment: by the two rows of small balls, we could establish the coordinate system of the swimming space of the diving beetle, the small balls were sprayed with different colors so that it could be easy to distinguish each ball, photosensitive resin material was used to print the 3D model. (For interpretation of the references to color in this figure legend, the reader is referred to the Web version of this article.)

The schematic diagram of the experimental apparatus is illustrated in Fig. 8(a). The transparent water tank was big enough for the diving beetles to swim freely, with a length and width of 29 cm and 12 cm. An adjustable LED lamp served as a light source, and a white cardboard was placed on the top of the glass tank to improve the brightness of the background. Two high-speed cameras were located at the side and bottom of the transparent glass tank respectively. The swimming videos were token at a rate of 1000 fps and a resolution of 1280 × 800. For analysis of the swimming videos, the video of the calibration frame (Fig. 8(b)) must be taken first. To ensure the accuracy of analysis, at least 11 balls were required to appear in the field of view. After taking the calibration frame video, the calibration frame should be taken away before capturing the diving beetle’s swimming videos. Both the calibration frame video and the diving beetle’s swimming video were imported together into SIMI Motion software for motion analysis.

**Fig. 9:**
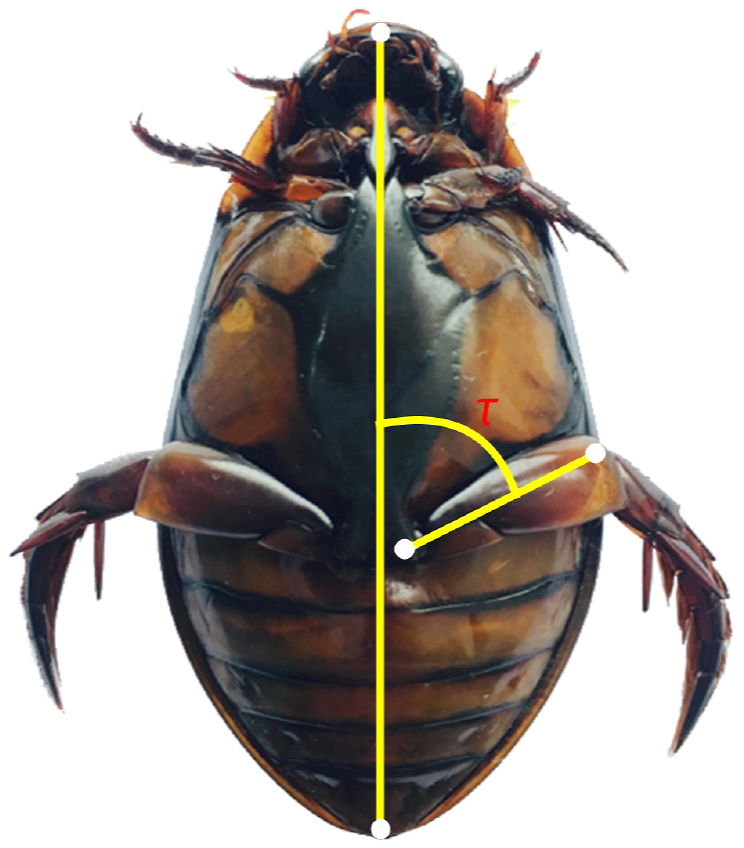
The diagram of the tracked point locations and the tracked angle. The angle *τ* is the angle between the diving beetle’s body axis and the femur.

The main swimming modes, including forwarding, turning and retreating, were captured in the experiments except for the turning mode. In this paper, we researched the dynamics of diving beetles while swimming in an approximate plane. Based on the 3D scanning model of the diving beetle, we obtained the position of the centroid, which was approximately in the middle of the diving beetle’s body axis. However, since it is difficult to track the centroid of the diving beetle in the swimming video, we selected the head and tail as the tracked points. The angle between the diving beetle’s body axis and the femur was chosen as the base for determining the power stroke or recovery stroke, as shown in Fig. 9.

Even though we chose a large enough transparent glass tank to ensure that the diving beetle could swim freely, it was still difficult to guarantee the appearance of an integrated swimming cycle in the video. And the swimming route of the diving beetle was random. We picked out two swimming videos that best correspond to the two swimming modes (forwarding and retreating, as shown in Supplementary Video S1 and Supplementary Video S2). To ensure accuracy in analysis, each swimming video was analyzed three times using SIMI Motion, and the motion data in the manuscript is the average of the three analysis results (as shown in Supplementary Data S2 and Supplementary Data S3).

Supplementary data related to this article can be found at https://doi.org/10.1016/j.heliyon.2023.e14200.

The following are the supplementary data related to this article:

Multimedia component 5 Multimedia component 5

Multimedia component 6 Multimedia component 6

### 4.3 Methods of solving the propulsion force and return resistance

The main task of this paper is to determine the propulsion force (during power stroke) and return resistance (during recovery stroke) of the diving beetle’s hind legs while swimming straight forward or backward underwater. These values were then used to calculate the propulsion efficiency of the diving beetle. The calculation of these forces follows Newton’s second law of motion, detailed below.

**Fig. 10:**
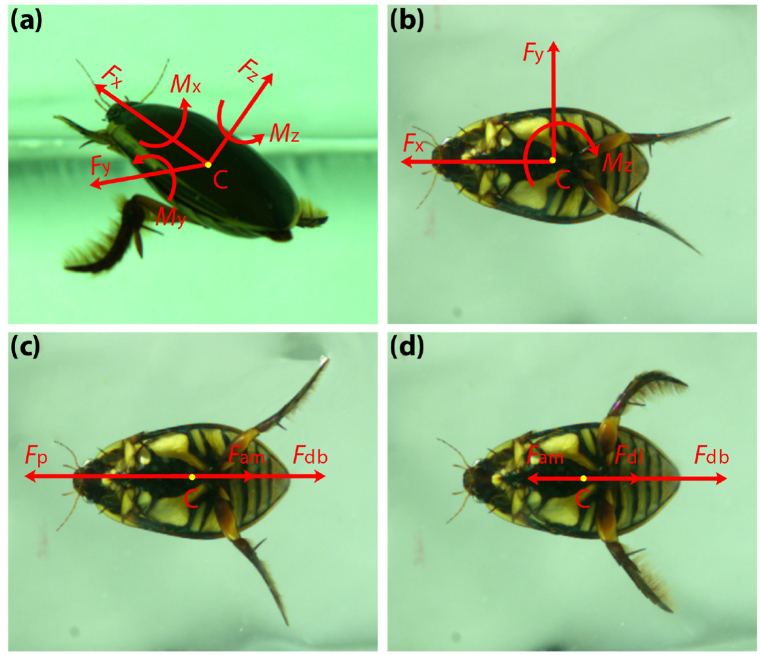
Force and torque diagrams at the centroid of the diving beetle while swimming. (a) Force and torque diagram at the centroid of the diving beetle while swimming in three dimensions, where x, y, and z axes are the directions from the diving beetle’s tail to head, right to left, and belly to back, respectively. (b) Force and torque diagram at the centroid of the diving beetle while moving in a plane. (c) Force and torque diagram at the centroid of the diving beetle during the power stroke of straight forward swimming. (d) Force and torque diagram at the centroid of the diving beetle during the recovery stroke of straight forward swimming.

When the diving beetle moves underwater three-dimensionally, the forces it receives can be simplified as three orthogonal forces and three torques acting on the centroid (Fig. 10(a)). However, in daily swimming motions, diving beetles usually move in a plane (the forces they received are shown in Fig. 10(b)). Since the movement of the two hind legs is symmetrical, they usually keep swimming in an approximately straight line if there are no obstacles in front of them. We separated straight forward swimming and straight backward swimming from the captured swimming videos of diving beetles and carried out a detailed study.

In the direction of the diving beetle’s body axis, the hydrodynamic forces acting on the diving beetle during the power stroke of straight forward or backward swimming mainly include the propulsion forces acting on the right and left hind legs (*F*_pr_ and *F*_pl_), the drag force acting on the body (*F*_db_), and the added mass force generated by the acceleration of the diving beetle (*F*_am_, if not negligible), as shown in Fig. 10(c). Based on Newton’s second law of motion, we could list Eq. (6).

(6) $$F_{p}-F_{\text{am}}-F_{\text{db}}=ma$$

where *F*_p_ is the sum of *F*_pl_ and *F*_pr_; *F*_am_ is the added mass force; *F*_db_ is the drag force acting on the body; *m* is the mass of the diving beetle; *a* is the acceleration of the diving beetle. All forces and acceleration only represent values in the direction of the diving beetle’s body axis.

Similarly, during the recovery stroke of straight forward or backward swimming, the hydrodynamic forces acting on the diving beetle include the drag forces acting on the right and left hind legs (*F*_dlr_ and *F*_dll_), the drag force acting on the body (*F*_db_), and the added mass force generated by the deceleration of the diving beetle (*F*_am_), as shown in Fig. 10(d). Based on Newton’s second law of motion, we could list Eq. (7).

(7) $$F_{\text{dl}}+F_{\text{db}}-F_{\text{am}}=ma$$

where *F*_dl_ is the sum of *F*_dlr_ and *F*_dll_; *F*_db_ is the drag force acting on the body; *F*_am_ is the added mass force; *m* is the mass of the diving beetle; *a* is the acceleration of the diving beetle. All the forces and acceleration only represent the values of them in the direction of the diving beetle’s body axis.

### 4.4 The calculation of the kinematic and kinetic parameters

From Eqs. (6), (7), for the calculation of *F*_p_ and *F*_dl_, the values of the kinematic variables velocity *v* and acceleration *a*, and the kinetic variables *F*_db_ and *F*_am_ must be solved. Their calculation methods will be explained in detail in the following contents.

#### 4.4.1 Movement velocity v and acceleration a at the centroid of the diving beetle

The swimming videos of the diving beetle captured by the high-speed camera system were processed using SIMI Motion software. The coordinates, velocities, and accelerations of the marker points in each frame were collected. The frame frequency of the videos was converted to 200 fps for the purpose of ensuring accuracy in the analysis of speed and acceleration. According to the obtained videos, it was found that the diving beetle could only maintain an approximately linear motion in daily swimming. This because the forces acting on the two hind legs are different to some degree. Since our main purpose is to determine the forces acting on the diving beetle’s hind legs in the direction of its body axis, the velocity *v* and acceleration *a* in the direction of the axis must also be calculated. Since the position of the centroid is approximately in the middle of the diving beetle’s body axis, movement velocity *v* and acceleration *a* at the centroid of the diving beetle could be calculated by Eqs. (8), (9), and the velocity *v* and acceleration *a* in the direction of the body axis could be calculated by Eqs. (10), (11).

(8) $$\left\{ \begin{array}{l} v_{cx}=\frac{v_{hx}+v_{tx}}{2}\\ v_{cy}=\frac{v_{hy}+v_{ty}}{2} \end{array}\right.$$

(9) $$\left\{ \begin{array}{l} a_{cx}=\frac{a_{hx}+a_{tx}}{2}\\ a_{cy}=\frac{a_{hy}+a_{ty}}{2} \end{array}\right.$$

(10) $$v_{cl}=v_{c}\cdot e_{l}=\left(v_{cx},v_{cy}\right)\cdot \frac{\pm \left(x_{h}-x_{t},y_{h}-y_{t}\right)}{\sqrt{{\left(x_{h}-x_{t}\right)}^{2}+{\left(y_{h}-y_{t}\right)}^{2}}}=\pm \frac{v_{cx}\left(x_{h}-x_{t}\right)+v_{cy}\left(y_{h}-y_{t}\right)}{\sqrt{{\left(x_{h}-x_{t}\right)}^{2}+{\left(y_{h}-y_{t}\right)}^{2}}}$$

(11) $$a_{cl}=a_{c}\cdot e_{l}=\left(a_{cx},a_{cy}\right)\cdot \frac{\pm \left(x_{h}-x_{t},y_{h}-y_{t}\right)}{\sqrt{{\left(x_{h}-x_{t}\right)}^{2}+{\left(y_{h}-y_{t}\right)}^{2}}}=\pm \frac{a_{cx}\left(x_{h}-x_{t}\right)+a_{cy}\left(y_{h}-y_{t}\right)}{\sqrt{{\left(x_{h}-x_{t}\right)}^{2}+{\left(y_{h}-y_{t}\right)}^{2}}}$$

where *v*_*cx*_ and *v*_*cy*_ are the velocities at the centroid of the diving beetle in the *x*-axis and *y*-axis directions, respectively; *v*_*hx*_, *v*_*hy*_ and *v*_*tx*_, *v*_*ty*_ are the head velocities and tail velocities in the *x*-axis and *y*-axis directions, respectively; *a*_*cx*_ and *a*_*cy*_ are the accelerations at the centroid of the diving beetle in the *x*-axis and *y*-axis directions, respectively; *a*_*hx*_, *a*_*hy*_ and *a*_*tx*_, *a*_*ty*_ are the head accelerations and tail accelerations in the *x*-axis and *y*-axis directions, respectively; ***e***_*l*_ is the unit vector of the body axis in the direction of motion, and it is “+” when the diving beetle swimming forward and “–” when the diving beetle swimming backward; *x*_*h*_, *y*_*h*_ and *x*_*t*_, *y*_*t*_ are the coordinates of the head and tail, respectively.

#### 4.4.2 Drag force acting on the diving beetle’s body (*F*_*db*_)

Like other ordinary rigid bodies, the drag force acting on the diving beetle’s body under the assumption that it swims at a constant speed usually depends on many factors, including the density and viscosity of the fluid and the geometry, surface characteristics, and velocity of the body. The dimensionless Reynolds number of the fluid plays a key role in determining the drag force [24], and is defined by Eq. (12).

(12) $$R=\frac{\rho dv}{\mu }$$

where *ρ* is the density of the fluid; *v* is the velocity of the body in the fluid; *μ* is the dynamic viscosity of the fluid; *d* is the characteristic length. As shown in Table 4, they are the *ρ* and *μ* values of liquid water at different temperatures [25].

**Table 4:** The *ρ* and *μ* values of liquid water at different temperatures.

Temperature (°C)	Density *ρ*, (kg·m^−3^)	Viscosity *μ*, (10^−6^ Pa·s)
20	998.2336	1002.0
25	997.0751	890.3
30	995.6783	797.5
35	994.0635	719.5

The minimum and maximum velocities of the diving beetle during forward swimming are 5.9 cm·s^−1^ and 13.2 cm·s^−1^, respectively, and 3.1 cm·s^−1^ and 23.2 cm·s^−1^ during backward swimming (as shown in Fig. 3). Under the conditions that the length of the diving beetle’s body (36.3 mm ± 0.67 mm) was chosen as the characteristic length and the temperature at 30 °C, the Reynolds numbers *R* range from 2674 to 5982 and 1405 to 10,514, respectively. Since these values are moderate, it can’t be determined whether the main component of *F*_db_ is viscous force or inertial force [24,[26], [27], [28]]. Finally, Eq. (13) and Eq. (14) were selected as the forms of *F*_db_ (the equation will be chosen based on the analysis results). Solving the drag coefficients *C*, *C*_1_, and *C*_2_ is the main task, which will be introduced in the next section.

(13) $$F_{\text{db}}=\frac{1}{2}\rho v^{2}CS_{W}$$

(14) $$F_{\text{db}}=C_{1}\mu vL+\frac{1}{2}\rho v^{2}C_{2}S_{W}$$

where *C*, *C*_1_, and *C*_2_ are drag coefficients and can be regarded as constants under a certain range of Reynolds number; *μ* is the dynamic viscosity of the water; *v* is the velocity of the body in the water; *L* is the length scale (36.3 mm); *ρ* is the density of the water; *S*_*W*_ is the frontal area of the diving beetle.

### 4.5 The added mass force *F*_*am*_

When two same rigid bodies move in liquid with the same velocity, the one with acceleration will experience a bigger drag force, and the difference between them is the so-called added mass force [20,29]. The reason for this phenomenon is that when a rigid body accelerates in the liquid, the surrounding liquid will also follow the acceleration. Consequently, the liquid inertial force will react to the rigid body, and it seems that the mass of the rigid body becomes larger. This added part is called added mass [30]. The relationship between the added mass force and added mass is shown in Eq. (15).

(15) $$F_{\text{am}}=m^{*}a$$

where m* is added mass, *a* is the acceleration of the rigid body, and the condition is that the fluid is stationary or flowing at a constant velocity.

In Eq. (15), the solution methods of added mass were introduced from the perspective of experiment and simulation in many papers [[31], [32], [33], [34], [35], [36]]. Since the diving beetle is so small that it is difficult to obtain the added mass through experimental means, CFD simulation method was chosen in this paper to solve the added mass. The specific processes are detailed below.

**Fig. 11:**
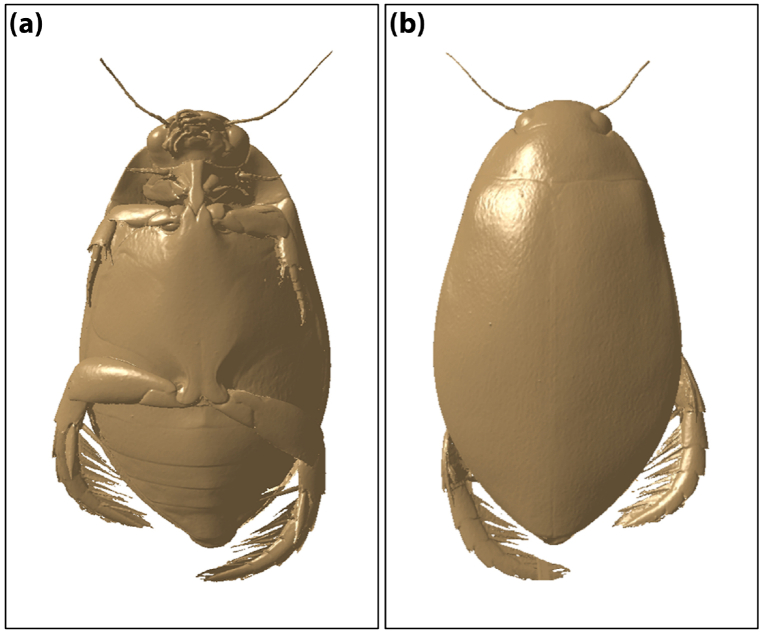
The 3D scanning model of the diving beetle. (a) The front side of the model. (b) The back side of the model.
(2) Fitting the curves of the drag force of water based on the least squares method. The independent variables are the velocity and acceleration of the diving beetle model (*v* and *a*), and the dependent variable is the drag force of water (*F*_dw_). The functions that need to be fitted are as Eq. (16) [20].

(1) Simulation of the acceleration process of the diving beetle was based on the 3D scanning model (Fig. 11(a) and Fig. 11(b), the simulation results are as shown in Supplementary Data S4).

(16) $$\left\{ \begin{array}{l} F_{\text{dw}}=F_{\text{db}}+F_{\text{am}}=\hat{a}v^{2}+\hat{b}v+\hat{c}a\;\left(a\right)\\ F_{\text{dw}}=F_{\text{db}}+F_{\text{am}}=\hat{d}v^{2}+\hat{e}a\;\left(b\right) \end{array}\right.$$

where $\hat{a}$, $\hat{b}$, $\hat{c}$, $\hat{d}$, and $\hat{e}$ are constants that need to be fitted; *v* and *a* are the velocity and acceleration of the diving beetle, respectively.

(3) According to the components of *F*_db_, we could get the solutions for *C*, *C*_1_, *C*_2_, and m* by Eqs. (17), (18), (19), (20).

(17) $$C_{1}=\frac{\hat{b}}{\mu L}$$

(18) $$C_{2}=\frac{2\hat{a}}{\rho S_{W}}$$

(19) $$C=\frac{2\hat{d}}{\rho S_{W}}$$

(20) $$m^{*}=\hat{c}\;\text{or}\;\hat{e}$$

## CRediT author statement

**D. Qi:** Conceived and designed the experiments; Analyzed and interpreted the data; Wrote the paper. **C. Zhang:** Conceived and designed the experiments; Contributed reagents, materials, analysis tools or data. **Z. Wu:** Analyzed and interpreted the data; Contributed reagents, materials, analysis tools or data. **C. Shen:** Conceived and designed the experiments; Contributed reagents, materials, analysis tools or data. **Y. Yue:** Performed the experiments. **L. Ren:** Conceived and designed the experiments; Contributed reagents, materials, analysis tools or data. **L. Yang:** Conceived and designed the experiments; Wrote the paper. All authors have read and approved the final submitted manuscript.

## Funding

This study was supported by the 10.13039/501100012166National Key Research and Development Program of China (grant number 2018YFA0703300), the 10.13039/501100001809National Natural Science Foundation of China (grant numbers 52275289, 51875243), and the Science and Technology Development Program of Jilin Province (grant number 20220508144RC).

## Data availability

All data generated or analyzed during this study are included in this published article (and its Supplementary Materials).

## Declaration of competing interest

The authors declare that they have no known competing financial interests or personal relationships that could have appeared to influence the work reported in this paper.

## References

[1] Ribera I., Foster G.N., Holt W.V. (1997). Functional types of diving beetle (Coleoptera: Hygrobiidae and Dytiscidae), as identified by comparative swimming behaviour. Biol. J. Linn. Soc..

[2] Whittlesey R.W. (2011). Wake-based unsteady modeling of the aquatic beetle Dytiscus marginalis. J. Theor. Biol..

[3] Sudo S., Tsuyuki K., Matsumoto T., Yoshikawa M., Watanabe M., Honda T. (2007). Biomimetic study of diving beetle robot propelled by alternating magnetic field. Int. J. Appl. Electromagn. Mech..

[4] Miller K.B., Bergsten J. (2016).

[5] Glaeser G., Nachtigall W. (2019). Signaling, swimming, flying, exploding. Evol. Funct. Biol. Macrostruct...

[6] Benetti C.J., Michat M.C., Ferreira N., Braga R.B., Megna Y.S., Toledo M. (2018). Thorp and Covich’s Freshwater Invertebrates.

[7] Sudo S., Yano T., Kan Y., Yamada Y., Tsuyuki K. (2006). Swimming behavior of small diving beetles. J. Adv. Sci..

[8] Voise J., Casas J. (2010). The management of fluid and wave resistances by whirligig beetles. J. R. Soc. Interface.

[9] Xu Z., Lenaghan S.C., Reese B.E., Jia X., Zhang M. (2012). Experimental studies and dynamics modeling analysis of the swimming and diving of whirligig beetles (Coleoptera: Gyrinidae). PLoS Comput. Biol..

[10] Kwak B., Bae J. (2017). Design of hair-like appendages and comparative analysis on their coordination toward steady and efficient swimming. Bioinspiration Biomimetics.

[11] Nachtigall W. (1981). Hydromechanics and biology. Biophys. Struct. Mech..

[12] Nachtigall W. (1961). Dynamics and energetics of swimming in water-beetles. Nature.

[13] Kim H.J., Jun B.H., Lee J. (2014).

[14] Kim H.J., Lee J. (2012). IEEE Int. Conf. Robot. Biomimetics.

[15] Alexander R.M. (2003). Principles of Animal Locomotion.

[16] Chen Y., Doshi N., Goldberg B., Wang H., Wood R.J. (2018). Controllable water surface to underwater transition through electrowetting in a hybrid terrestrial-aquatic microrobot. Nat. Commun..

[17] Kwak B., Bae J. (2017). Toward fast and efficient mobility in aquatic environment: a robot with compliant swimming appendages inspired by a water beetle. J. Bionic Eng..

[18] Kim H., Lee J. (2017). Design, swimming motion planning and implementation of a legged underwater robot (CALEB10: D.BeeBot) by biomimetic approach. Ocean Eng..

[19] Sudo S. (2010). Biomimetics, Learning from Nature.

[20] Brennen C.E. (1982). Technical Report–CR82.010.

[21] Ribera I., Nilsson A.N. (1995). Morphometric patterns among diving beetles (Coleoptera: Noteridae, Hygrobiidae, and Dytiscidae). Can. J. Zool..

[22] Hughes G.M. (1958). The co-ordination of insect movements III. Swimming in Dytiscus, Hy-drophilus and a Dragonfy Nymph. J. Exp. Biol..

[23] Feldmann D., Das R., Pinchasik B.E. (2020). How can interfacial phenomena in nature inspire smaller robots. Adv. Mater. Interfac..

[24] Long L.N., Weiss H. (1999). The velocity dependence of aerodynamic drag: a primer for mathematicians. Am. Math. Mon..

[25] Kestin J., Sokolov M., Wakeham W.A. (1978). Viscosity of liquid water in the range of -8 °C to 150 °C. J. Phys. Chem. Ref. Data.

[26] Daniel T.L. (1984). Unsteady aspects of aquatic locomotion. Am. Zool..

[27] Clemente C.J., Richards C. (2013). Muscle function and hydrodynamics limit power and speed in swimming frogs. Nat. Commun..

[28] Gal J.M., Blake R.W. (1988). Biomechanics of frog swimming: I. Estimation of the propulsive force generated by hymenochirus boettgeri. J. Exp. Biol..

[29] Levy S., Wilkinson J.P.D. (1975). Structural Mechanics in Reactor Technology.

[30] Raza N., Mehmood I., Rafiuddin H., Rafique M. (2012).

[31] Caspersen C., Berthelsen P.A., Eik M., Pâkozdi C., Kjendlie P.L. (2010). Added mass in hu-man swimmers: age and gender differences. J. Biomech..

[32] Salibindla A.K.R., Masuk A.U.M., Ni R. (2021). Experimental investigation of the acceleration statistics and added-mass force of deformable bubbles in intense turbulence. J. Fluid Mech..

[33] Liu L., Sun M. (2018). The added mass forces in insect flapping wings. J. Theor. Biol..

[34] Mohaghegh F., Udaykumar H.S. (2017). Comparison of sharp and smoothed interface methods for simulation of particulate flows II: inertial and added mass effects. Comput. Fluids.

[35] Wang Z., Fan D., Triantafyllou M.S. (2021). Illuminating the complex role of the added mass during vortex induced vibration. Phys. Fluids.

[36] Javanmard E., Mansoorzadeh S., Mehr J.A. (2020). A new CFD method for determination of translational added mass coefficients of an underwater vehicle. Ocean Eng..

